# The effects of prednisolone treatment on serological responses and lipid profiles in Ethiopian leprosy patients with Erythema Nodosum Leprosum reactions

**DOI:** 10.1371/journal.pntd.0007035

**Published:** 2018-12-28

**Authors:** Edessa Negera, Melaku Tilahun, Kidist Bobosha, Saba M. Lambert, Stephen L. Walker, John S. Spencer, Abraham Aseffa, Hazel M. Dockrell, Diana N. Lockwood

**Affiliations:** 1 London School of Hygiene and Tropical Medicine (LSHTM), London, United Kingdom; 2 Armauer Hansen Research Institute (AHRI), Addis Ababa, Ethiopia; 3 Department of Microbiology, Immunology & Pathology, Colorado State University, Fort Collins, CO, United States of America; Swiss Tropical and Public Health Institute, SWITZERLAND

## Abstract

**Background:**

Erythema nodosum leprosum (ENL) is a systemic inflammatory complication occurring mainly in patients with lepromatous leprosy (LL) and borderline lepromatous leprosy (BL). Prednisolone is widely used for treatment of ENL reactions. However, it has been reported that prolonged treatment with prednisolone increases the risk for prednisolone-induced complications such as osteoporosis, diabetes, cataract and arteriosclerosis. It has been speculated that perhaps these complications result from lipid profile alterations by prednisolone. The effects of extended prednisolone treatment on lipid profiles in ENL patients have not been studied in leprosy patients with ENL reactions. Therefore, in this study we conducted a case-control study to investigate the changes in lipid profiles and serological responses in Ethiopian patients with ENL reaction after prednisolone treatment.

**Methods:**

A prospective matched case–control study was employed to recruit 30 patients with ENL and 30 non-reactional LL patient controls at ALERT Hospital, Ethiopia. Blood samples were obtained from each patient with ENL reaction before and after prednisolone treatment as well as from LL controls. The serological host responses to PGL-1, LAM and Ag85 *M*. *leprae* antigens were measured by ELISA. Total cholesterol (TC), triglyceride (TG), high density lipoprotein (HDL) and low density lipoprotein (LDL) were measured by spectrophotometric method.

**Results:**

The host antibody response to *M*. *leprae* PGL-1, LAM and Ag85 antigens were significantly reduced in patients with ENL reactions compared to LL controls after treatment. Comparison between patients with acute and chronic ENL showed that host-response to PGL-1 was significantly reduced in chronic ENL after prednisolone treatment. Untreated patients with ENL reactions had low lipid concentration compared to LL controls. However, after treatment, both groups had comparable lipid profiles except for LDL, which was significantly higher in patients with ENL reaction. Comparison within the ENL group before and after treatment showed that prednisolone significantly increased LDL and HDL levels in ENL patients and this was more prominent in chronic ENL than in acute patients with ENL.

**Conclusion:**

The significantly increased prednisolone-induced LDL and TG levels, particularly in patients with chronic ENL reactions, is a concern in the use of prednisolone for extended periods in ENL patients. The findings highlight the importance of monitoring lipid profiles during treatment of patients to minimize the long-term risk of prednisolone-induced complications.

## Author summary

Erythema Nodosum Leprosum (ENL) reaction is a severe multisystem immune-mediated complication of lepromatous and borderline leprosy. It causes high morbidity and mortality and usually requires urgent medical attention. Although thalidomide is an effective drug for ENL treatment, it is not available in many leprosy endemic countries including Ethiopia. Prednisolone is widely used for treatment of ENL reactions but its efficacy is less than 40%. As a result, patients with ENL reactions receive Prednisolone for prolonged periods. However, it has been reported that prolonged treatment with prednisolone increases the risk for prednisolone-induced complications such as osteoporosis, diabetes, cataract and arteriosclerosis. It has been hypothesized that perhaps these complications result from changes in lipid concentration due to prednisolone. Therefore, this study was aimed to determine changes in lipid profiles in patients with ENL reactions. We found that prednisolone treatment not only alters lipid concentrations in patients with ENL reactions but also reduced the antibody responses to *M*. *leprae* antigens. Our result has shown that prednisolone treatment has increased low and high lipoproteins in patients with ENL reactions. We also found that use of prednisolone for prolonged time in chronic ENL was correlated with increased triglycerides (TG) and low density lipoproteins (LDL) showing the need for monitoring lipid profiles during prednisolone treatment of these patients to avoid the risks associated with increased TG and HDL such as diabetes and hypertension.

## Introduction

Leprosy is a disease caused by *Mycobacterium leprae* which mainly affects the skin and the peripheral nerves[1]. Depending on the host immune response, the disease manifests with a spectrum of five relatively distinct clinical pictures: localized tuberculoid leprosy (TT), three forms of borderline leprosy (BT, BB, BL) and the generalized lepromatous leprosy (LL) based on the Ridley-Jopling (RJ) classification [2]. In addition to the five clinical forms, most leprosy patients develop reactions called type-1 and type-2 leprosy reactions [3]. Leprosy reactions are immune-mediated incidents of acute or sub-acute inflammation and are the main complications of the disease leading to permanent disability. Type-2 or Erythema Nodosum Leprosum reaction (ENL) is an immune-mediated inflammatory complication, occurring in about 50% of LL and 10% of borderline lepromatous leprosy (BL) patients[4, 5]. ENL occurs as an acute episode but can develop into a chronic phase or can be recurrent [6]. It involves multiple organs and manifests as a systemic illness [7]. The occurrence of crops of tender erythematous skin lesions is the clinical diagnostic feature of ENL[8]. Accurate laboratory confirmation for ENL is not yet available. Potential biomarkers related to inflammatory cytokines such as TNF-α [9], reduced regulatory T-cells[10], increased levels of neutrophils infiltration [11], mycobacterial cell-wall and protein antigens [12] have been investigated.

Identification and characterization of *M*. *leprae* specific antigens for accurate and reliable diagnosis of leprosy and leprosy reactions is a major priority in leprosy research. Phenolic glycolipid-1 (PGL-1) is a surface glycolipid in *M*. *leprae* which is believed to interact with the host immune cells [13]. PGL-I has been implicated in the tropism of *M*. *leprae* for Schwann cells, through specific binding to laminin, and is reported to play an important role in down-regulation of the inflammatory immune response and inhibition of dendritic cell maturation and activation, thereby facilitating the persistence of *M*. *leprae* [14].

ENL is treated with prednisolone or with thalidomide. Thalidomide is not imported in most leprosy endemic countries such as Ethiopia and is restricted due to its potential for birth defects in humans when taken during pregnancy. Prednisolone is an immunosuppressive drug used for the treatment of chronic inflammatory diseases. In the majority of patients, ENL is a chronic condition requiring prolonged immunosuppression [15]. Although the adverse effects of prednisolone treatment in patients with ENL has not been well studied, one study has reported significant mortality and morbidity associated with prolonged administration of prednisolone in patients with chronic ENL in Ethiopia [15]. A meta- analysis study reported that prolonged treatment with prednisolone increases the risk for osteoporosis and fracture, thinning and bruising of the skin, risk of developing cataracts and arthrosclerosis [16].

Steroid induced diabetes is increasingly frequent in patients receiving prolonged steroid treatment [17]. Among 20 patients with ENL reaction who had received prednisolone treatment in a recent study, 5 (25%) were reported to have developed diabetes mellitus while 7(35%) developed hypertension ([17]. Similarly, a significant proportion (23.5%) of leprosy patients treated with prednisolone for reactions developed steroid induced diabetes mellitus in India [18]. Several studies suggest that the adverse effects of prednisolone could result from altered lipid profiles in patients with inflammatory diseases [16–18]. In patients with adrenal insufficiency, prednisolone increased LDL levels significantly and is a recognized risk factor for cardiovascular disease in these patients ([19]. Similar effects have also been reported in Rheumatoid Arthritis [20] and Systemic Lupus Erythematosus [21].

However, the effects of prolonged (months to several years) prednisolone treatment on lipid profiles in patients with ENL reactions have not been investigated although all Ethiopian leprosy patients with ENL reactions are being treated with prednisolone. We conducted a case-control follow-up study to investigate the changes in lipid profiles and evaluate the serological responses in patients with ENL reactions after prednisolone treatment. We also investigated the changes in lipid profiles in patients with acute and chronic ENL.

## Materials and methods

### Ethics statement

The study was reviewed and approved by the AHRI/ALERT Ethics review committee, (P032/12) and the National Research Ethics Review Committee, Ethiopia (#310/450/06) as well as by the Institutional Ethical Committee of the London School of Hygiene and Tropical Medicine, UK, (#6391). Written informed consent was obtained from all study participants before enrolment. Children under 18 years were excluded from the study. All personal information was kept confidential and data was analysed anonymously.

### Study design and sample size determination

A prospective matched case-control study with follow-up after the initiation of prednisolone treatment was used to recruit 30 untreated patients with ENL reactions (i.e. patients newly presenting with reactions who have not yet received prednisolone treatment) and 30 non-reactional LL patient controls between December 2014 and January 2016 at ALERT Hospital, Ethiopia. Sample size was calculated assuming a constant probability of exposure thorough the pool of controls. Sample size was obtained by using the G* power 3.1.7 software with the input parameters: α = 0.05, power (β) = 0.8.

### Patient recruitment and blood sample collection

All patients recruited into this study were attending the ALERT Hospital, Addis Ababa, Ethiopia. All study patients were recruited to the study between December 2014 and January 2016.The patients were classified clinically on the leprosy spectrum based on the Ridley-Jopling (RG) classification schemes[2]. ENL was clinically diagnosed when a patient with BL or LL leprosy had painful crops of tender cutaneous erythematous skin lesions and systemic features of disease often fever, neuritis and bone pain with or without other accompanying clinical symptoms such as neuritis, joint pain, bone tenderness, orchitis, iritis, oedema malaise, anorexia and lymphadenopathy [5]. New ENL was defined as the occurrence of ENL for the first time in a patient with LL or BL. The nature of ENL was defined as acute for a single episode lasting less than 24 weeks while on corticosteroids treatment, recurrent if a patient experienced a second or subsequent episode of ENL occurring 28 days or more after stopping treatment for ENL and chronic if occurring for 24 weeks or more during which a patient required ENL treatment either continuously or where any treatment free period had been 27 days or less [8, 15]. Lepromatous leprosy was clinically diagnosed when a patient had widely disseminated nodular lesions with ill-defined borders and bacterial index (BI) above 2 [8, 15]. Patients were recruited prospectively. Patients with other leprosy clinical spectrum such as tubeculoid (TT), borderlines and type 1 reactions were excluded from the study. Pregnant and lactating mothers, anaemic patients, patients with concomitant sever conditions such as TB, HIV/AIDS, cardiac and renal problems and diabetes mellitus were excluded. Patients receiving any medication other than MDT were also excluded from the study.

Patients with ENL were treated according to the World Health Organization (WHO) treatment guideline with steroids that initially consisted of 40 mg oral prednisolone daily and the dose was tapered by 5 mg every fortnight for 24 weeks. All patients were given WHO-recommended leprosy multidrug treatment (MDT). From each patient 10 mL venous blood sample was obtained before treatment and after prednisolone treatment of patients with ENL reactions. A fifteen day prednisolone treatment free period was used to obtain blood samples for the after treatment sample [22, 23].

### Serological response quantification by ELISA

The levels of anti ND-O-BSA (PGL-1), LAM and Ag85 antibodies were measured by ELISA in the plasma samples of patients with ENL and LL controls before and after treatment as previously described [24]. Recombinant protein Ag85, ND-O-BSA (PGL-1), and LAM were coated onto high-affinity polystyrene flat-bottom 96-well ELISA plates (Dynex Technologies, Chantilly, VA) using 50ng/well in 100μl buffer, pH 9.6 at 4°C overnight. Unbound antigen was washed away using PBS, pH 7.4, containing 1% Bovine serum albumin (BSA) and 0.05% Tween 80 (blocking buffer) six times. Serial 2-fold dilutions of plasma samples from1:100 to 1:10^6^ diluted in 100 μl blocking buffer were added to the wells and incubated for 2 h at room temperature. After incubation with the primary antibody, the wells were washed six times as described above and followed by the addition of 100 μl of a 1:5,000 dilution of the secondary anti-human polyvalent antibody (Sigma) for 2 h. Following washing the wells with PBS six times, 100 μl of *p*-nitrophenylphosphate substrate (Kirkegaard and Perry Labs, Gaithersburg, MD) was added. The absorbance at 405 nm was read using a VersaMax Pro plate reader (Molecular Devices, Sunnyvale, CA) at 15min. BSA was used as negative control and all tested samples had an optical density (OD) value less than 0.2 for BSA. An OD value above 1.5 was assigned as high response for PGL^-^1 and LAM, and 1.0 for Ag85.

### Lipid profile analysis

Serum levels of total cholesterol (TC), triglyceride (TG), high density lipoprotein (HDL) and low density lipoprotein (LDL) were measured by the spectrophotometric method using a fully automated clinical chemistry analyser Mindray BS-120 (Mindray, China) according to the recommendations of the manufacturers [25]. Analysis for serum samples for TC, TG, HDL and LDL was carried out at the AHRI clinical laboratory. For external quality control 20 randomly selected samples were analysed for total lipid profiles at ALERT clinical chemistry laboratory using A25 Biosystem chemistry analyser (Oxford, UK). Total triglycerides were determined using commercial reagents as described by [26]. Total high density lipoprotein and low density lipoprotein levels were determined by enzymatic clearance method [27]. TC concentration was estimated using the cholesterol oxidase/peroxidase method [28].

### Statistical analysis

#### Lipid profiles

Unpaired *t*-test was used to compare the relative concentration of each lipid profile in patients with ENL and LL controls. For comparing the lipid concentration in patients with ENL before and after treatment, a paired t-test was used. Comparison between acute and chronic ENL was made using unpaired t-test. Results are presented as mean ± standard error of the mean (SE) with a P-value cut-off of <0.05.

#### Antibody responses

Serological response data were analysed with two-tailed Mann- Whitney U test using STATA 14 version 2 (San Diego California USA). Median estimator with inter-quartile range was used for result presentation. For all data, graphs were produced by GraphPad Prism version 5.01 for Windows (GraphPad Software, San Diego California USA).

## Results

### Study patients’ clinical background

Thirty LL patients with ENL reaction (15 acute ENL and 15 chronic ENL) and 30 LL patient controls without ENL reaction were recruited into the study. In patients with ENL, the male to female ratio was 2:1; and patients had a median age of 27.5 [range: 18–56] years. In patients with non-reactional LL controls, the male to female ratio was 3:1 with a median age of 25.0 [range: 18–60] years. None of the patients with ENL reactions had received prednisolone at the time of enrolment to the study. At time of recruitment, 20 ENL patients were previously untreated with MDT. After the first blood sample collection, ENL patients were immediately started on regular treatment according to national and WHO guidelines.

### Prednisolone reduced antibody response to *M*. *leprae* PGL-1 antigen in patients with ENL reactions

**Fig 1:**
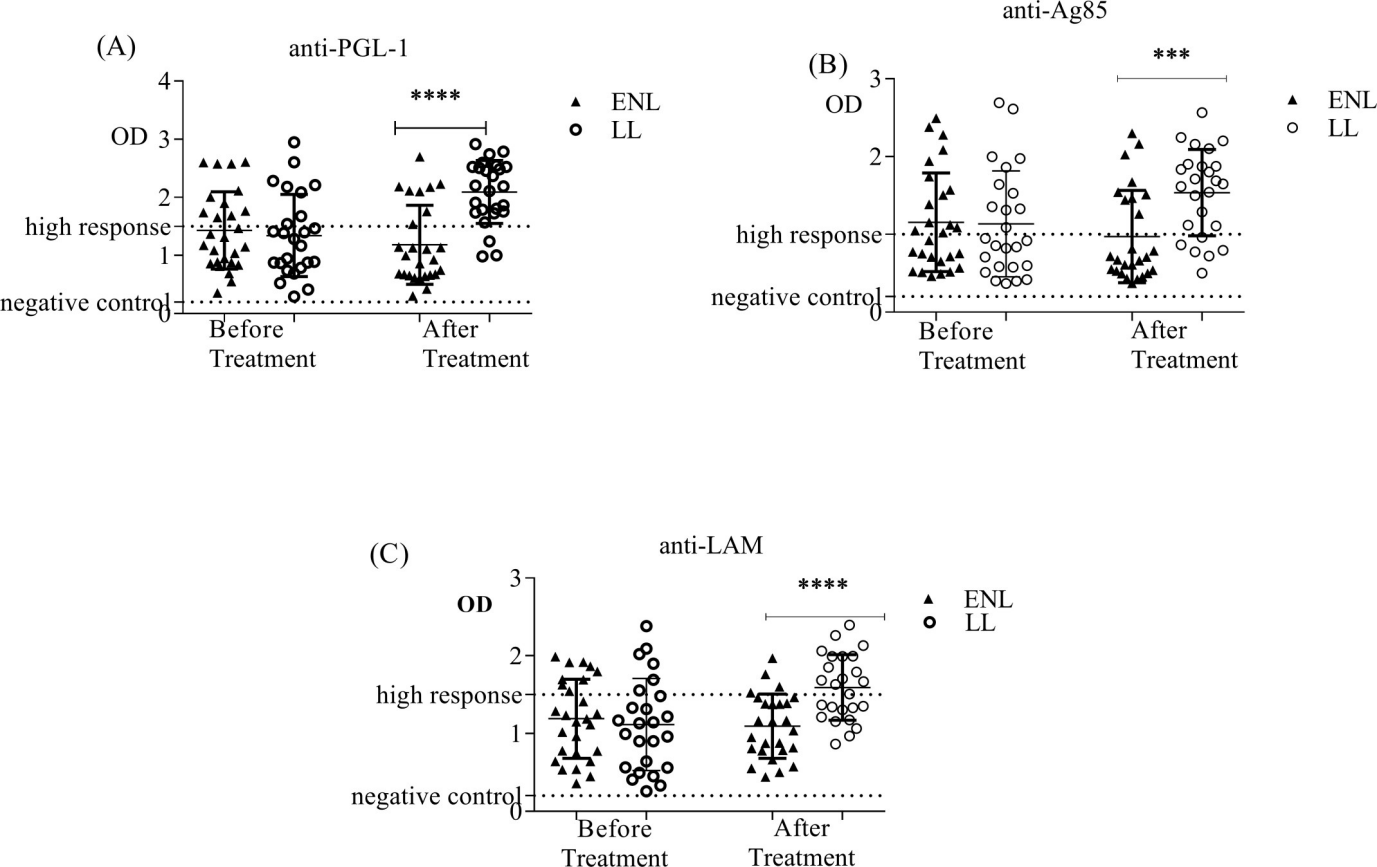
Serological responses to antigens in patients with ENL compared to LL patient controls before and after prednisolone treatment of patients with ENL reaction. (A): response to *M*. *leprae* PGL-1 antigen; (B): response to *M*. *leprae* Ag85 antigen; (C): response to *M*. *leprae* LAM antigen; Number of ENL = LL = 25; *Statistical test*: *Mann-Whitney unpaired test (U)*. *** P≤0.001; **** P<0.*0001*. Error bars show median ± interquartile range.

The concentration of anti-PGL-1 antibody was not significantly different in patients with ENL and LL controls before treatment of ENL cases with prednisolone (Fig 1A). Similarly, the levels of anti -LAM and anti-Ag85 antibody titres were not significantly different in patients with ENL and LL controls before treatment (Fig 1B and 1C). However, after treatment, patients with ENL had significantly lower anti-PGL-1 antibody titre than LL patient controls (p < 0.0001). Similarly, anti -LAM and anti-Ag85 antibody titres were significantly low in patients with ENL compared to LL controls after prednisolone treatment of ENL cases (p < 0.0001) (Fig 1A–1C).

**Fig 2:**
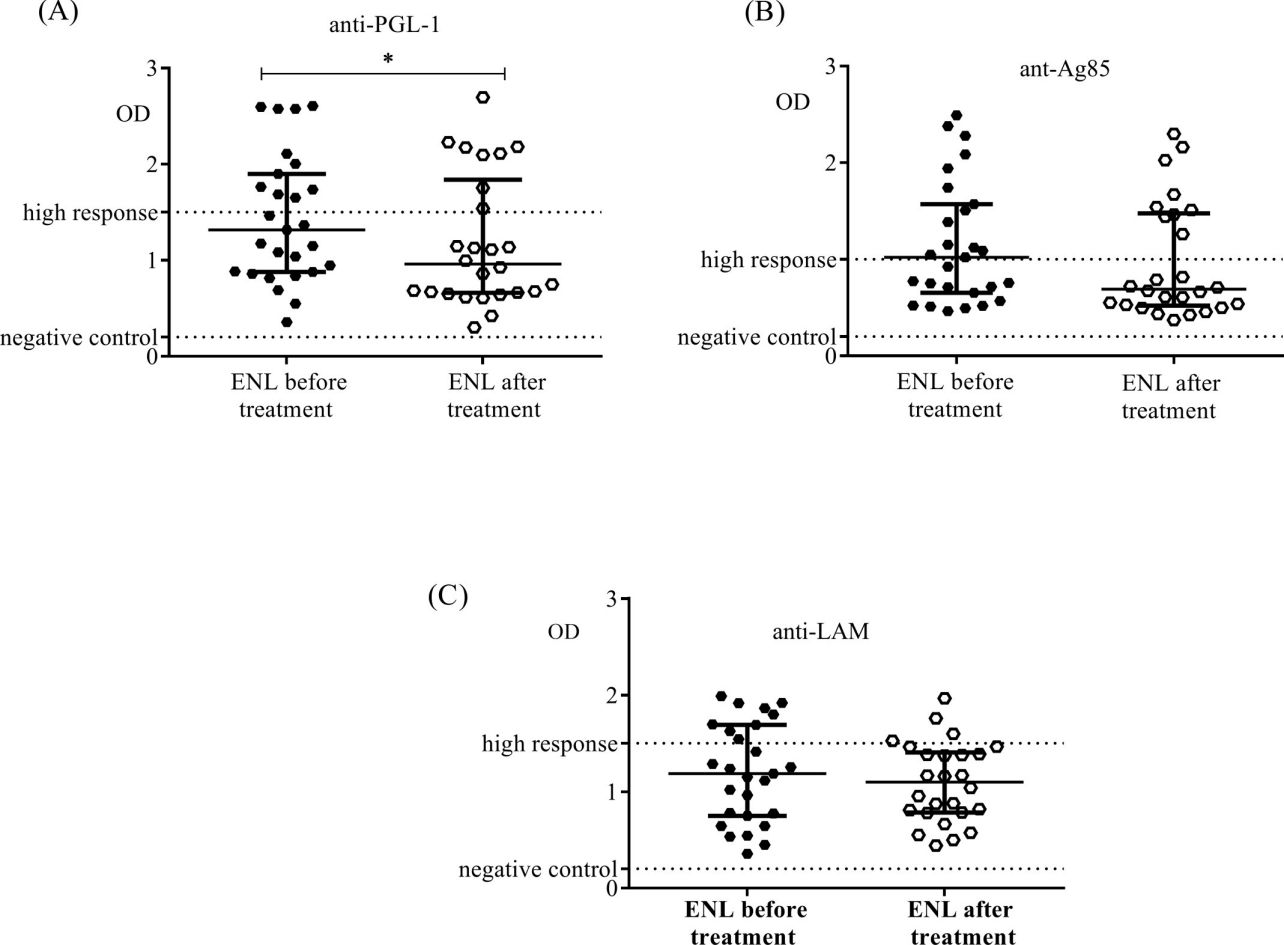
Serological responses to antigens in patients with ENL before and after prednisolone treatment (A): response to *M*. *leprae* PGL-1 antigen; (B): response to *M*. *leprae* Ag85 antigen; (C): response to *M*. *leprae* LAM antigen; Number of ENL = LL = 25; *Statistical test*: *Mann-Whitney unpaired test (U)*. * P≤0.*05;* Error bars show median ± interquartile range.

Comparison within ENL groups showed after prednisolone treatment of ENL the response of plasma samples to PGL-1 antigen was significantly reduced (P = 0.01). On the other hand, the response of plasma samples from ENL cases to LAM and Ag85 antigens did not change after prednisolone treatment (Fig 2A–2C).

### Patients with chronic ENL reactions had significantly low serum response to *M*. *leprae* PGL-1 antigen

**Fig 3:**
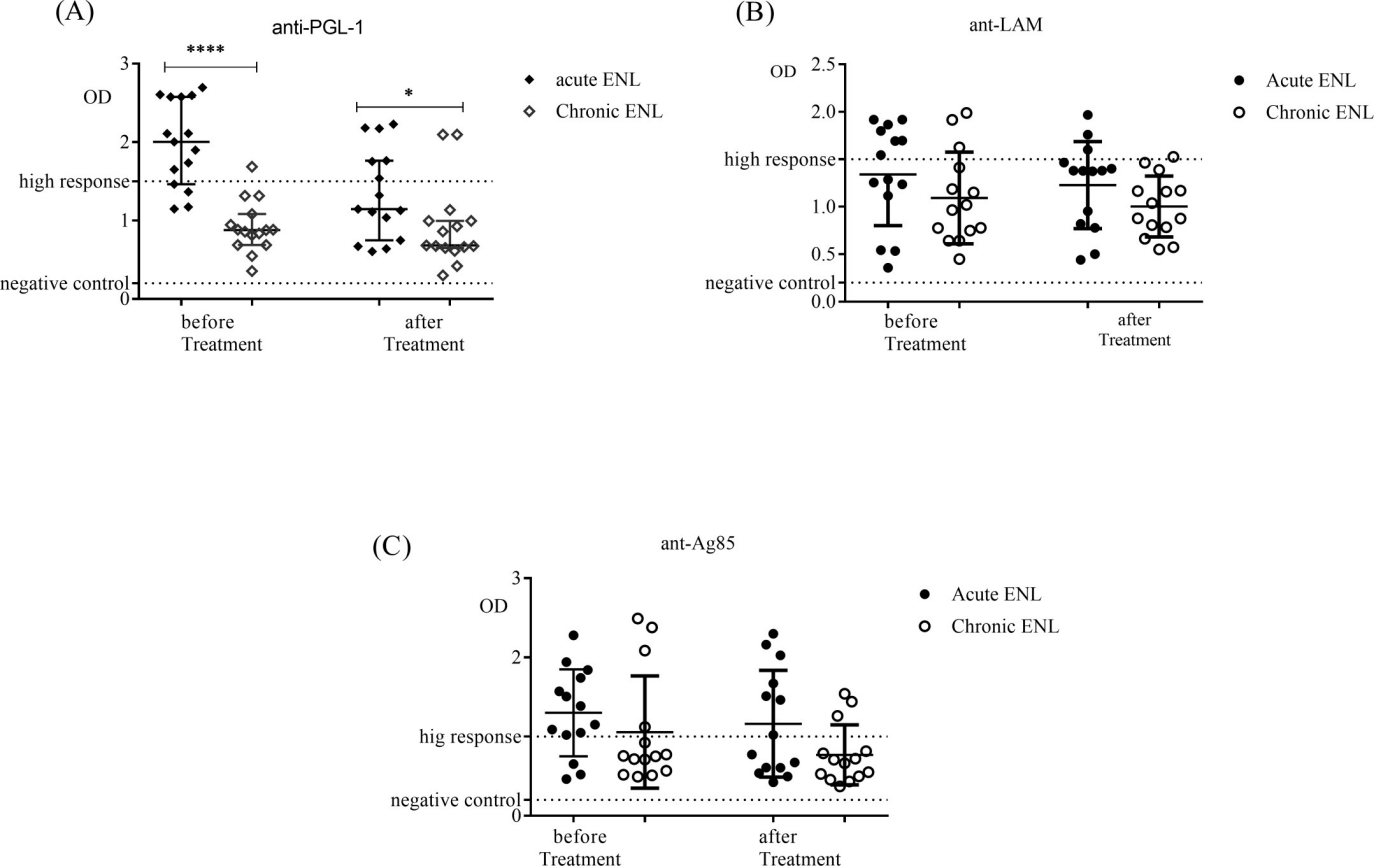
Serological responses to antigens in patients with acute ENL (n = 15) compared with patients with chronic ENL (n = 15) before and after treatment. (A): response to *M*. *leprae* PGL-1 antigen; (B): response to *M*. *leprae* Ag85 antigen; (C): response to *M*. *leprae* LAM antigen; *Statistical test*: *Mann-Whitney unpaired test (U)*. * P≤0.05; ** P<0.*005*. Error bars show median ± interquartile range.

ENL patients were classified clinically into acute and chronic ENL. The serological responses in serum samples from acute and chronic ENL were compared before and after prednisolone treatment of ENL cases. Serum samples from untreated acute ENL patients had significantly higher responses (p<0.0001) to *M*. *leprae* PGL-1 antigen than serum samples from untreated chronic ENL patients (Fig 3A). Although the response to PGL-1 was significantly reduced in both groups after prednisolone treatment, the reduction was greater in chronic ENL (Fig 3A).

On the other hand, the serological response in serum samples from untreated acute and chronic ENL patients to LAM antigens was not significantly different. The serum response to LAM antigen did not change after prednisolone treatment in both acute and chronic ENL (Fig 3B). Response to Ag85 in untreated acute and chronic ENL patients was also not significantly different. Similarly, after prednisolone treatment, the response to Ag85 did not change in either acute or chronic ENL (Fig 3C).

**Fig 4:**
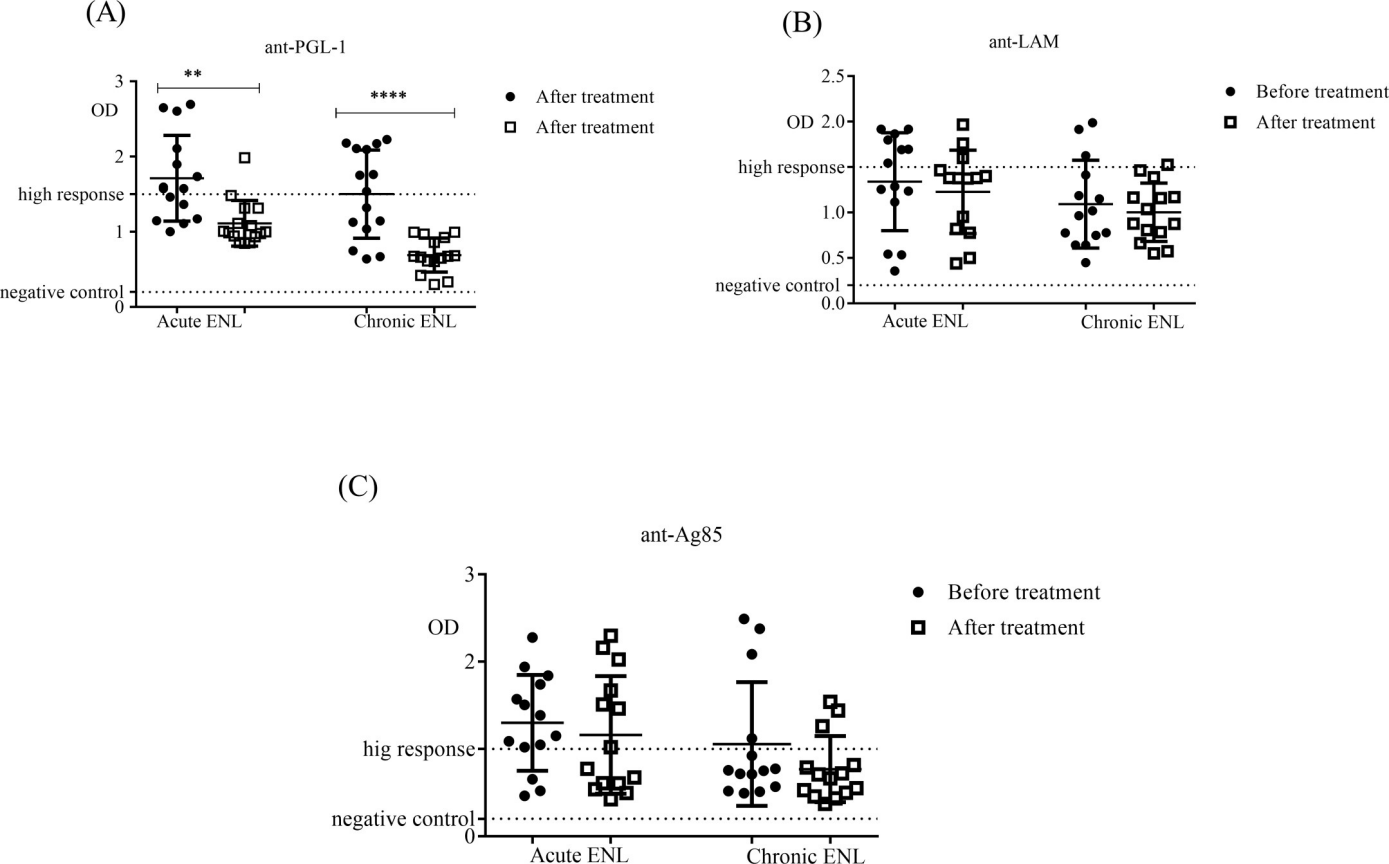
Serological responses to antigens in patients with acute and chronic ENL before and after prednisolone treatment. (A): response to *M*. *leprae* PGL-1 antigen; (B): response to *M*. *leprae* LAM antigen; (C): response to *M*. *leprae* Ag85 antigen; Number of ENL = LL = 25; Statistical test: Mann-Whitney unpaired test *(U)*. * P≤0.05; **** P<0.*0001*. Scatter plots show median ± interquartile range.

Comparison within acute and chronic ENL cases before and after prednisolone treatment showed that prednisolone treatment significantly reduced antibody response to PGL-1 in patients with acute and chronic ENL. However, prednisolone greatly affected the serological response to PGL-1 in chronic ENL cases compared to in acute ENL cases (Fig 4A). On the other hand, prednisolone treatment did not affect serological response to LAM and Ag85 in acute as well as chronic ENL cases (Fig 4B and 4C).

### Untreated patients with ENL reactions had low lipid profiles compared to LL controls

**Fig 5:**
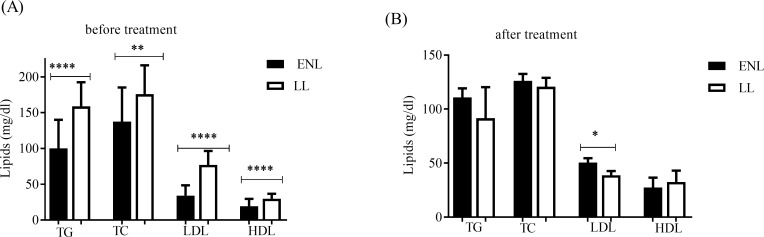
Lipid profile of patients with ENL and LL controls before and after prednisolone treatment of ENL cases. (A): before treatment; (B): after treatment. Number of ENL = LL = 30; *Statistical test*: *unpaired t- test*. * P≤0.05; *** P<0.*001*, **** P<0.*0001*. Error bars show mean ± standard error of the mean. TG = Triglycerides, TC = total cholesterol, LDL = low-density lipoprotein, HDL = high density lipoprotein.

We analysed the serum lipid profile in 30 patients with ENL cases and 30 LL patient controls before and after prednisolone treatment of ENL cases. The levels of serum triglycerides were low (99.95 ± 6.046 SE mg/dl) in patients with ENL reactions compared to LL patient controls (158.7 ± 7.394 SE mg/dl) before treatment (P<0.0001) (Fig 5A). After prednisolone treatment of ENL cases, the serum concentration of triglyceride was slightly increased in ENL patients to 110.8 ± 8.318 SE mg/d and reduced to 91.50 ± 5.876 SE mg/d in LL controls though the difference was not statistically significant (Fig 5B).

The concentration of serum low-density lipoprotein (LDL) in untreated patients with ENL reactions was below half (34.05mg/dl ± 2.186SE) that of the corresponding concentration (77.05mg/dl ± 4.225SE) in LL patient controls (P<0.0001) (Fig 5A). Interestingly, after treatment, the concentration of LDL was significantly increased (50.52mg/dl ± 4.016SE) in patients with ENL reactions while it was decreased by half (38.75mg/dl ± 3.910SE) in LL patient controls (P = 0.0413) (Fig 5B).

Untreated ENL patients had lower serum total cholesterol (137.4mg/dl ± 7.210SE) than LL controls (175.8mg/dl ± 8.805SE) (P = 0.0023). However, after treatment, the total cholesterol concentration decreased to 126.2mg/dl ± 6.333SE and 120.7mg/dl ± 8.250SE in ENL patients and LL controls respectively (Fig 5B). Similarly, untreated ENL patients had a significantly lower serum concentration of high density lipoproteins (HDL) (9.20mg/dl ± 1.565SE) compared to LL controls (29.76mg/dl ± 1.491SE) (P<0.0001). After treatment, in patients with ENL reactions, the concentration of HDL was increased to 27.48mg/dl ± 1.822SE with the corresponding value of 32.58mg/dl ± 2.131SE in LL controls but the difference was not statistically significant (Fig 5B).

### Prednisolone significantly increased low and high-density lipoproteins in patients with ENL reactions

**Fig 6:**
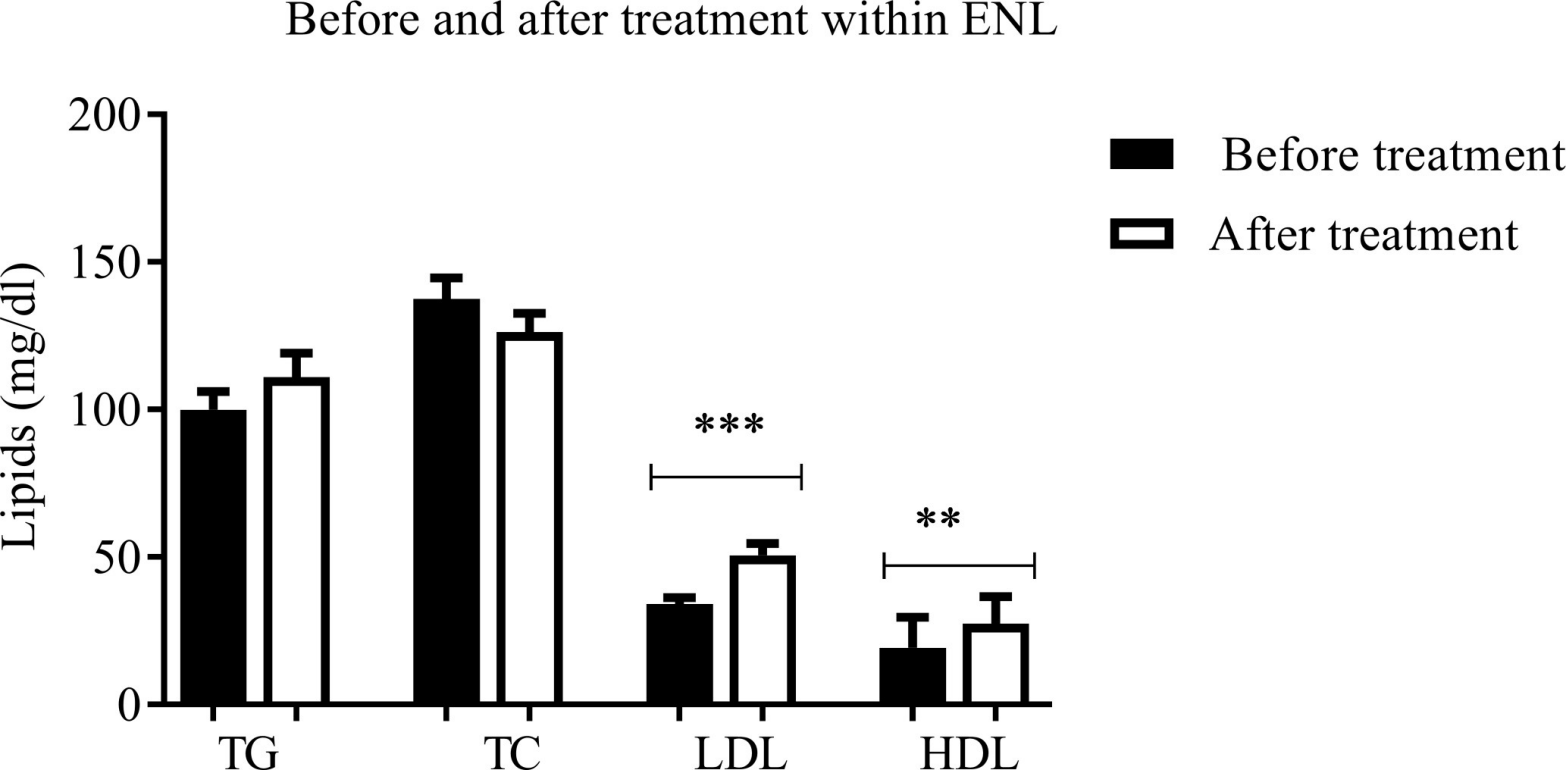
Lipid profile within ENL before and after treatment. Number of ENL = 30; *Statistical test*: *unpaired t- test*. ** P<0.*005*, *** P<0.*001*. Error bars show mean ± standard error of the mean. TG = Triglycerides, TC = total cholesterol, LDL = low-density lipoprotein, HDL = high-density lipoprotein.

Comparison within ENL before and after prednisolone treatment has shown that prednisolone treatment increased the mean serum LDL concentration from 34.05mg/dl ± 2.186SE to 50.52mg/dl ± 4.016SE in patients with ENL reactions and the difference was statistically significant (P = 0.0002). Similarly, the mean HDL concentration was significantly increased from 19.20mg/dl ± 1.565SE to 27.48mg/dl ± 1.822SE after prednisolone treatment of patients with ENL reactions (P = 0.0014) (Fig 6).

On the other hand, although the mean serum concentration of triglycerides (TG) increased from 99.95mg/dl ± 6.046SE to 110.8mg/dl ± 8.318SE after prednisolone treatment, this did not reach statistical significance. Unlike the other lipid profiles, the mean serum concentration of total cholesterol (TG) showed a decreasing tendency from 137.4mg/dl ± 7.210SE to 126.2mg/dl ± 6.333 SE after prednisolone treatment although the difference was not statistically significant (Fig 6).

### Prednisolone alters serum concentration of triglycerides and low-density lipoproteins in chronic ENL

**Fig 7:**
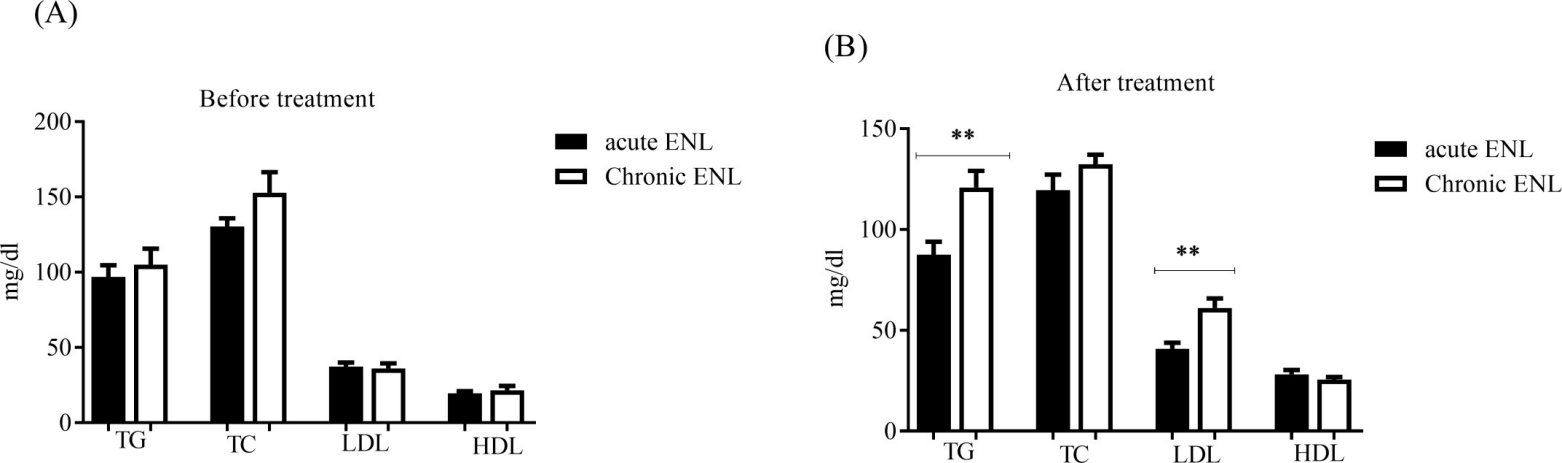
Lipid profiles before and after prednisolone treatment of acute ENL patients (n = 15) compared with chronic ENL patients (n = 15). (A): before treatment; (B): after treatment.; *Statistical test*: *unpaired t- test*. ** P<0.*005*. Error bars show mean ± standard error of the mean. TG = Triglycerides, TC = total cholesterol, LDL = low-density lipoprotein, HDL = high-density lipoprotein.

The effects of prednisolone treatment on patients with acute and chronic ENL reactions were compared before and after treatment. Untreated patients with acute ENL reactions had slightly lower serum triglycerides concentrations (96.7mg/dl±7.918 SE) than untreated patients with chronic ENL reactions (105.0 mg/dl± 10.65SE) and the difference was not statistically significant (Fig 7A). However, after prednisolone treatment, in patients with chronic ENL reactions the serum concentrations of TG was significantly increased to 120.8mg/dl ± 8.224SE and was greater than in patients with acute ENL reactions (87.40mg/dl ± 6.496SE, (P = 0.0029) (Fig 7B). Untreated patients with acute ENL reactions had comparable serum LDL (37.20 mg/dl± 2.755SE and 36.15mg/dl ± 3.188E respectively) (Fig 7A). Interestingly, after prednisolone treatment serum LDL was significantly increased in patients with chronic ENL reactions (61.00mg/dl ± 4.855SE) compared with patients with acute ENL reactions (40.85mg/dl ± 2.910SE, P = 0.001) (Fig 7B).

On the other hand, untreated patients with acute and chronic ENL reactions had similar serum total cholesterol (130.4mg/dl ± 5.306SE and 152.8mg/dl ± 13.74SE respectively) (Fig 7A). After prednisolone, treatment the concentration of total cholesterol in patients with acute ENL reactions (119.4mg/dl ± 7.741SE) and chronic ENL reactions (132.3mg/dl ± 4.754SE) was not statistically significantly changed (Fig 7B). Similarly, untreated patients with chronic and acute ENL reactions had 19.40mg/dl ± 1.538SE and 21.65mg/dl ± 2.811SE serum HDL concentrations respectively and the difference between the two groups was not statistically significant. After prednisolone treatment, HDL concentration was increased to 28.15mg/dl ± 2.214SE in patients with acute ENL reactions and to 25.60mg/dl ± 1.177SE in patients with chronic ENL reactions but the difference was not statistically significant (Fig 7B). Hence, prednisolone increased serum triglycerides and low-density lipoprotein largely in patients with chronic ENL reaction than in patients with acute ENL reactions.

**Fig 8:**
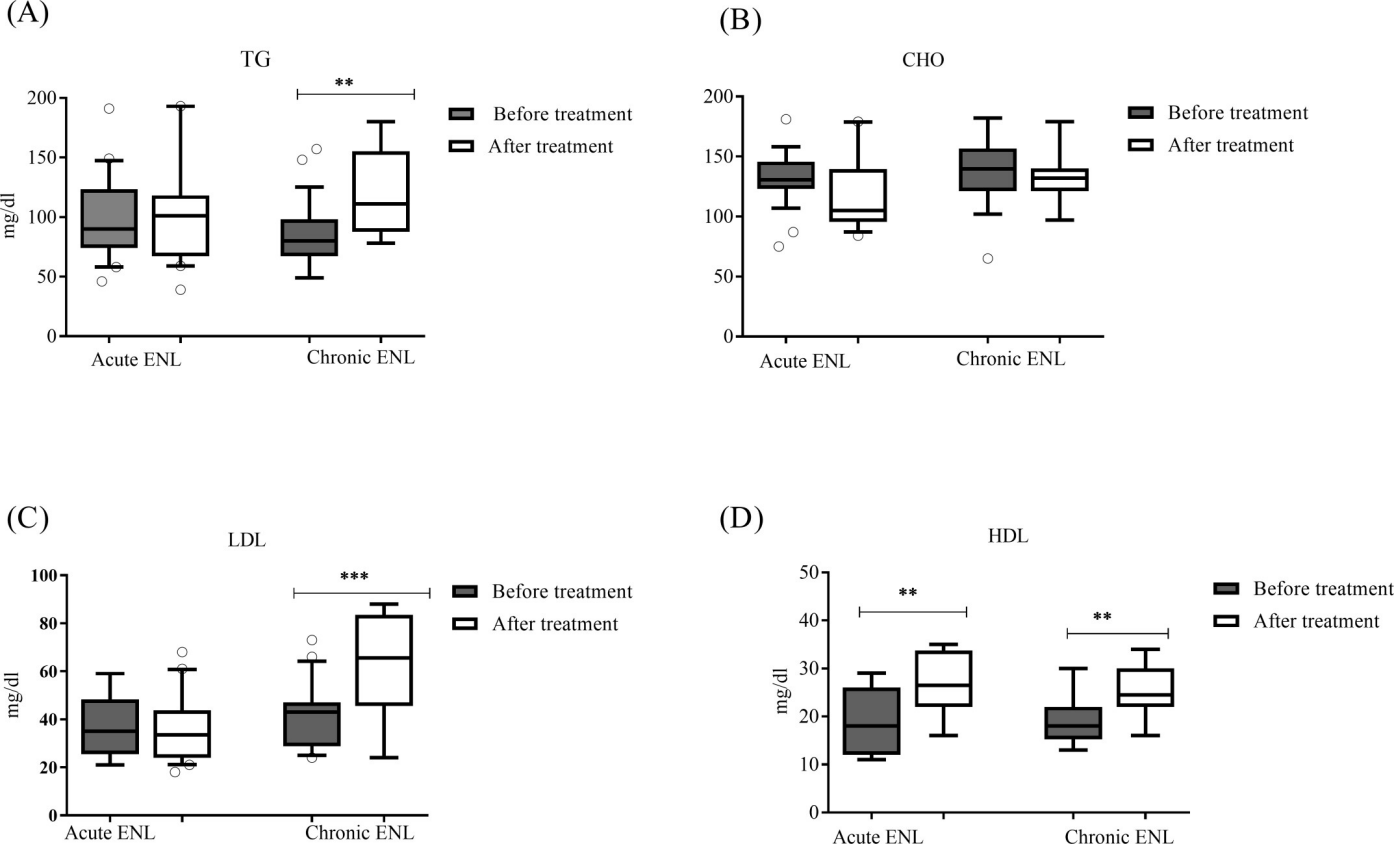
Lipid profiles before and after prednisolone treatment of acute and chronic ENL patients before and after prednisolone treatment. (A): Triglycerides; (B): Total cholesterol; (C): low-density lipoprotein; (D): High-density lipoprotein; Number of ENL = LL = 30; *Statistical test*: *unpaired t- test*. ** P<0.005; ***P<0.*001*. Box and whiskers show median ± interquartile range. TG = Triglycerides, TC = total cholesterol, LDL = low density lipoprotein, HDL = high density lipoproteins.

We also compared the lipids concentration in acute and chronic ENL patients before and after prednisolone treatment (Fig 8A–8D). Prednisolone treatment did not change the concentrations of triglycerides, total cholesterol and low-density lipoprotein in patients with acute ENL. However, the concentration of high-density lipoprotein was significantly increased in these patients after prednisolone treatment. On the other hand, prednisolone treatment significantly increased the concentrations of triglycerides, high and low density lipoprotein in patients with chronic ENL (Fig 8A–8C and 8D).

## Discussion

### Serological response

Identification and characterization of *M*. *leprae* specific antigens is among the major goals to be attained by researchers for accurate and reliable diagnosis of leprosy and leprosy reactions. The anti-PGL-1 antibody levels were not significantly different in patients with ENL (OD = 1.430 ± 0.1281) and LL controls (1.341 ± 0.1415) (P>0.05) before treatment. However, after treatment the level of anti-PGL-1 was significantly decreased (OD = 1.183 ± 0.1333) in patients with ENL compared to LL patient controls (OD = 2.091 ± 0.1081) (p < 0.0001). A study in Brazilian leprosy patients including 5 untreated ENL cases, 13 non- reactional LL patients and 13 other clinical forms of leprosy had reported that the levels of anti-PGL-1 were not significantly different in ENL and LL patients [29] which is in agreement with the present result. The finding of lower level of anti-PGL-1 in ENL cases than in the corresponding LL controls after prednisolone treatment of ENL patients in this study could be explained by the effect of prednisolone treatment on immune response [30]. Previous studies have shown that the level of anti-PGL-1 was significantly decreased in ENL patients treated with prednisolone compared to LL patients [31].

When acute and chronic ENL cases were compared, acute ENL cases had higher levels of anti-PGL-1 antibodies than chronic ENL cases before treatment (P≤0.001). Similar findings had been reported in an earlier study [32]. The differences in the levels of antibody response to PGL-1 in acute and chronic cases could probably reflect the higher rate of PGL-1 synthesis in acute ENL cases than in chronic ENL cases. PGL-1 is synthesised by viable and actively dividing *M*. *leprae* [14, 33]. After treatment, the levels of antibody responses to PGL-1 still remain lower in chronic ENL cases than in acute ENL cases and this could be due to the continue effect of ongoing prednisolone treatment in patients with chronic ENL.

Similar to anti-PGL-1, the levels of anti-LAM serum antibodies were not significantly different in patients with ENL (OD = 1.191 ± 0.09790) and LL controls (OD = 1.116 ± 0.1183) before treatment (P>0.05). However, after prednisolone treatment of ENL cases, the levels of anti-LAM were lower in ENL cases (OD = 1.095 ± 0.08101) than in LL controls (OD = 1.592 ± 0.08426) (P≤0.0001). The reduction of the level of antibody response to LAM in ENL cases after treatment could be due to the effect of prednisolone treatment as previously reported [34]

Antigen 85 (Ag85) complex proteins are major secretory products of *Mycobacterium*. Like for LAM, similar serum levels of anti-Ag85 antibodies were measured in the plasma samples from patients with ENL (OD = 1.154 ± 0.1218) and LL controls (OD = 1.134 ± 0.1360). After prednisolone treatment of ENL cases, the level of anti-Ag85 was lower in ENL cases (OD = 0.9713 ± 0.1164) than in in LL controls (OD = 1.536 ± 0.1108) (P≤0.001). The differences in the levels of antibody response to Ag85 after treatment in the two groups might be due to the effect of prednisolone treatment of ENL cases. However, this assumption should be further investigated and supported by definitive evidence.

### Lipid profiles

Despite their beneficial effects, long-term use of corticosteroids is generally associated with severe metabolic side effects including steroid-induced diabetes, muscle atrophy and disorders in lipid metabolism which limit their therapeutic usefulness [35]. Studies have shown that lipid concentrations are altered in leprosy across the spectrum and some researchers suggested the alteration in the lipid profile could be useful as a diagnostic tool for leprosy [36]. We have shown that untreated patients with ENL had lower TG, LDL and HDL than non-reactional LL patient controls (P ≤0.05). It has been shown that infection and inflammation are both associated with marked changes in lipid and lipoprotein metabolism. In addition to lipid transport, lipoproteins participate in innate immunity[37].Therefore, the findings of decreased serum lipid and lipoproteins in patients with ENL reactions could be due to the utilization of various types of lipids and lipoproteins in the process of inflammation as these patients are suffering from inflammatory complication of leprosy. Triglycerides are a major component of very-low-density lipoproteins (VLDL) and serve as a source of energy. During inflammation, TG provide lipid substrates for the activated immune system [37]. Cholesterol may also be used for lymphocyte activation and proliferation. Furthermore, infection is often associated with cellular injury and areas of injury may need extra cholesterol for new membrane synthesis [38].

Following prednisolone treatment of ENL cases, serum lipid levels increased significantly. The serum concentration of TG, TC and HDL in treated ENL was comparable with the corresponding values obtained for LL controls. However, LDL was significantly increased in patients with ENL reactions after they were treated with prednisolone compared to LL controls. Studies have shown that prednisolone leads to an adverse lipid profile with increased TC and LDL levels [19]. The significant increase of LDL in patients with ENL reactions after treatment with prednisolone would predict the possibility of prednisolone-induced complications in these patients although it needs further investigation.

Comparison within ENL groups before and after prednisolone treatment has shown that prednisolone treatment significantly increased the mean serum LDL concentration from 34.05mg/dl ± 2.186SE to 50.52mg/dl ± 4.016SE in patients with ENL reactions. Likewise, the mean HDL concentration was significantly increased from 19.20mg/dl ± 1.565SE to 27.48mg/dl ± 1.822SE after prednisolone treatment of patients with ENL reactions. Although prednisolone mediated adverse plasma lipid profiles are well documented, the mechanisms behind such changes are still not clear. Plasma cholesterol level is mainly regulated in the liver. The hepatic low-density lipoprotein receptor (LDLR), which contributes up to 80% of LDL clearance from the plasma in various animal species, affects both the rates of formation and clearance of LDL[39]. *In vitro* and *in vivo* studies have shown that prednisolone-mediated decreases in hepatic LDLR mRNA and activity, resulting in decreased binding and degradation of LDL in both humans and rats [39, 40]. Hence, the finding of increased serum concentration of LDL and HDL in patients with ENL reaction after prednisolone treatment shows the potential risk of prednisolone for LDL/HDL induced complications such as cardiovascular and hepatic complications.

We also analysed the lipid profile data to see the effect of prednisolone in patients with acute and chronic ENL. Interestingly our data show that prednisolone significantly alters serum concentration of triglycerides and low-density lipoproteins more so in chronic ENL than in patients with acute ENL. In patients with chronic ENL reactions, serum concentration of TG was significantly increased to 120.8mg/dl ± 8.224SE compared to patients with acute ENL reactions (87.40mg/dl ±6.496SE). Similarly, after prednisolone treatment, serum LDL was significantly increased in patients with chronic ENL reactions (61.00mg/dl ± 4.855SE) compared with patients with acute ENL reactions (40.85mg/dl ± 2.910SE). These differences in TG and LDL could be explained by the fact that patients with chronic ENL reaction receive more prednisolone for extended periods than patients with acute ENL.

### Conclusion

In conclusion, the significantly increased prednisolone-induced LDL and TG levels, particularly in patients with chronic ENL reactions calls for great caution when prednisolone is used for treatment of ENL reactions. However, to describe the long-term impact of prednisolone treatment on the lipid profiles of ENL patients we did not follow the patients after they completed their treatment. Hence, our finding does not account for whether prednisolone treatment causes temporary or permeant lipid profiles alteration in ENL patients. We recommend that lipid levels should be monitored and managed in patients with both acute and chronic ENL to minimize the long-term risk of prednisolone-induced complications. We also recommend that future research should focus on developing alternative and safe treatment options for ENL reaction.

## References

[1] Lockwood D., *Leprosy* *In*: Burns DA, Breathnach SM, Cox NH, Griffiths CEM, *editors* *Rook’s Textbook of Dermatology*. Oxford: Blackwell Publishing, 2004 7th ed 2: p. 2004. p. 29.

[2] Ridley D.S. and Jopling W.H., Classification of Leprosy according to immmunity: Five group system. Inter J lepr other Micobacterial Diseases, 1966 34(3). 5950347

[3] Lockwood D.N., et al, Clinical features and outcome of reversal (type 1) reactions in Hyderabad, India. International journal of leprosy and other mycobacterial diseases: official organ of the International Leprosy Association, 1993 61(1): p. 8–15. 8326184

[4] Van Veen N., et al, Interventions for erythema nodosum leprosum (Review). Cochrane Database of Systematic Reviews, 2009 3: p. No.: CD006949. 10.1002/14651858.CD006949.pub2 PMC11663503 19588412

[5] Pocaterra L., et al, Clinical Course of Erythema Nodosum Leprosum: An 11-year Chohort Study in Hyderabad, India. Am. J. Trop. Med. Hyg., 2006 74(5): p. 868–879. 16687695

[6] Walker S.L., et al, ENLIST 1: An International Multi-centre Cross-sectional Study of the Clinical Features of Erythema Nodosum Leprosum. PLoS Negl Trop Dis, 2015 9(9): p. e0004065 10.1371/journal.pntd.0004065 26351858 PMC4564249

[7] Walker S.L., Waters M.F., and Lockwood D.N., The role of thalidomide in the management of erythema nodosum leprosum. Lepr Rev, 2007 78(3): p. 197–215. 18035771

[8] Negera E., et al, Clinico-pathological features of erythema nodosum leprosum: A case-control study at ALERT hospital, Ethiopia. PLOS Neglected Tropical Diseases, 2017 11(10): p. e0006011 10.1371/journal.pntd.0006011 29028793 PMC5656324

[9] Moraes, et al, Cytokine mRNA Expression in Leprosy: a Possible Role for Interferon-γ and Interleukin-12 in Reactions (RR and ENL). Scandinavian Journal of Immunology, 1999 50(5): p. 541–549. 10564558 10.1046/j.1365-3083.1999.00622.x

[10] Negera E., et al, T-cell regulation in Erythema Nodosum Leprosum. PLOS Neglected Tropical Diseases, 2017 11(10): p. e0006001 10.1371/journal.pntd.0006001 28991896 PMC5648259

[11] Mabalay M.C., et al, THE HISTOPATHOLOGY AND HISTOCHEMISTRY OF ERYTHEMA NODOSUM LEPROSUM. International Journal of Leprosy, 1965 33: p. 28–49. 14282354

[12] Andreoli A., et al, Changes in circulating antibody levels to the major phenolic glycolipid during erythema nodosum leprosum in leprosy patients. Int J Lepr Other Mycobact Dis, 1985 53(2): p. 211–217. 3894538

[13] Schlesinger L.S. and Horwitz M.A., Phenolic glycolipid-1 of Mycobacterium leprae binds complement component C3 in serum and mediates phagocytosis by human monocytes. J Exp Med, 1991 174(5): p. 1031–8. 1940785 10.1084/jem.174.5.1031 PMC2118995

[14] Spencer J.S. and Brennan P.J., The role of Mycobacterium leprae phenolic glycolipid I (PGL-I) in serodiagnosis and in the pathogenesis of leprosy. Lepr Rev, 2011 82(4): p. 344–57. 22439275

[15] Walker S.L., et al, The Mortality Associated with Erythema Nodosum Leprosum in Ethiopia: A Retrospective Hospital-Based Study. PLoS Negl Trop Dis, 2014 8(3): p. e2690 10.1371/journal.pntd.0002690 24625394 PMC3953021

[16] Lipworth B.J., Systemic adverse effects of inhaled corticosteroid therapy: A systematic review and meta-analysis. Archives of Internal Medicine, 1999 159(9): p. 941–955. 10326936 10.1001/archinte.159.9.941

[17] Hwang J.L. and Weiss R.E., Steroid-induced diabetes: a clinical and molecular approach to understanding and treatment. Diabetes/metabolism research and reviews, 2014 30(2): p. 96–102. 10.1002/dmrr.2486 24123849 PMC4112077

[18] Papang R., et al, A study of steroid-induced diabetes mellitus in leprosy. Indian J Lepr, 2009 81(3): p. 125–9. 20509340

[19] Quinkler M., et al, Prednisolone is associated with a worse lipid profile than hydrocortisone in patients with adrenal insufficiency. Endocrine Connections, 2016. 10.1530/EC-16-0081 PMC5148794 27864317

[20] Gail K., et al, Associations of Hydroxychloroquine Use With Lipid Profiles in Rheumatoid Arthritis: Pharmacologic Implications. Arthritis Care & Research, 2014 66(11): p. 1619–1626. 24692402 10.1002/acr.22341

[21] Mohammad M.I., et al, Serum Lipid Profiles in Pediatric Systemic Lupus Erythematosus Patients: A Study from Bangladesh. American Journal of Clinical and Experimental Medicine, 2015 3(4): p. 255–259

[22] Magee M.H., et al, Prednisolone pharmacokinetics and pharmacodynamics in relation to sex and race. Journal of clinical pharmacology, 2001 41(11): p. 1180–1194. 11697751 10.1177/00912700122012733 PMC4207281

[23] Negera E., et al, The Effects of Prednisolone Treatment on Cytokine Expression in Patients with Erythema Nodosum Leprosum Reactions. Frontiers in Immunology, 2018 9(189). 10.3389/fimmu.2018.00189 PMC5811481 29479352

[24] Spencer J.S. and Brennan P., J., The Role of Mycobacterium leprae Phenolic Glycolipid I (PGL-I) in Serodiagnosis and in the Pathogenesis of Leprosy. Lepr Rev 2011 82: p. 344–357. 22439275

[25] Mehta R., et al, Serum lipid profile in patients with oral cancer and oral precancerous conditions. Dental Research Journal, 2014 11(3): p. 345–350. 10.4103/1735-3327.135889 25097644 PMC4119367

[26] Fossati P. and Prencipe L., Serum triglycerides determined colorimetrically with an enzyme that produces hydrogen peroxide. Clin Chem, 1982 28(10): p. 2077–80. 6812986

[27] Allen J.K., et al, An enzymic and centrifugal method for estimating high-density lipoprotein cholesterol. Clin Chem, 1979 25(2): p. 325–7. 215349

[28] Kayamori Y., et al, Endpoint Colorimetric Method for Assaying Total Cholesterol in Serum with Cholesterol Dehydrogenase. Clinical Chemistry, 1999 45(12): p. 2158 10585348

[29] Silva R.V.G., et al, Correlation between therapy and lipid profile of leprosy patients: is there a higher risk for developing cardiovascular diseases after treatment? Infectious Diseases of Poverty, 2017 6: p. 82 10.1186/s40249-017-0295-1 28457229 PMC5410692

[30] Oehling A.G., et al, Suppression of the immune system by oral glucocorticoid therapy in bronchial asthma. Allergy, 1997 52(2): p. 144–54. 9105518 10.1111/j.1398-9995.1997.tb00968.x

[31] Raju R., et al, Serological responses to prednisolone treatment in leprosy reactions: study of TNF-α, antibodies to phenolic glycolipid-1, lipoarabinomanan, ceramide and S100-B. Lipids in Health and Disease, 2014 13: p. 119–119. 10.1186/1476-511X-13-119 25070345 PMC4124507

[32] Bhoopat L., et al, Studies of Human Leprosy Lesions In Situ Using Suction-Induced Blisters: Cell Changes with IgM Antibody to PGL-1 and Interleukin-2 Receptor in Clinical Subgroups of Erythema Nodosum Leprosum. Asian Pacific Journal of Allergy and Immunology, 1991: p. 107–119. 1807258

[33] Lobato J., et al, Comparison of three immunological tests for leprosy diagnosis and detection of subclinical infection. Lepr Rev, 2011 82(4): p. 389–401. 22439279

[34] Raju R., et al, Serological responses to prednisolone treatment in leprosy reactions: study of TNF-α, antibodies to phenolic glycolipid-1, lipoarabinomanan, ceramide and S100-B. Lipids in Health and Disease, 2014 13(1): p. 119. 25070345 10.1186/1476-511X-13-119 PMC4124507

[35] Hazra A., et al, Modeling of Corticosteroid Effects on Hepatic Low-Density Lipoprotein Receptors and Plasma Lipid Dynamics in Rats. Pharmaceutical research, 2008 25(4): p. 769–780. 10.1007/s11095-007-9371-8 17674160 PMC4196440

[36] Ghulam S., et al, Comparative Study of Lipid Profile in Multibacillary and Paucibacillary Leprosy Patients. JBUMDC 2016 6(1): p. 43–46.

[37] Oiknine J. and Aviram M., Increased susceptibility to activation and increased uptake of low density lipoprotein by cholesterolloaded macrophages. Arterioscler.Thromb., 1992 I. 12: p. 745–753. 1591234 10.1161/01.atv.12.6.745

[38] Cuthbert J.A. and Lipsky P.E., Regulation of lymphocyte proliferation by cholesterol: the role of endogenous sterol metabolism and low density lipoprotein receptors. Int. J. Tissue React, 1987 9: p. 447–457. 3448024

[39] Bilheimer D.W., Regulation of LDL receptors in vivo. Agents Actions Suppl, 1984 16: p. 191–203. 6592957 10.1007/978-3-0348-7235-5_22

[40] Staels B., et al, Variable effects of different corticosteroids on plasma lipids, apolipoproteins, and hepatic apolipoprotein mRNA levels in rats. Arterioscler Thromb, 1991 11(3): p. 760–9. 1903065 10.1161/01.atv.11.3.760

